# Omega-3 Fatty Acids and Vitamin D in Cardiology

**DOI:** 10.1155/2012/729670

**Published:** 2012-12-31

**Authors:** Norbert Güttler, Kirila Zheleva, Mariana Parahuleva, Ridvan Chasan, Mehmet Bilgin, Christiane Neuhof, Mehmet Burgazli, Bernd Niemann, Ali Erdogan, Andreas Böning

**Affiliations:** ^1^Department of Cardiology and Angiology, University Hospital Giessen, 35390 Giessen, Germany; ^2^Department of Radiology, Bezmialem University of Istanbul, Istanbul, Turkey; ^3^Department of Cardiology, University of Giessen, 35390 Giessen, Germany; ^4^Department of Internal Medicine, University of Giessen, 35390 Giessen, Germany; ^5^Department of Cardiac Surgery, University of Giessen, 35390 Giessen, Germany; ^6^Department of Cardiovascular Surgery, University Hospital Giessen, 35390 Giessen, Germany

## Abstract

Dietary modification and supplementation play an increasingly important role in the conservative treatment of cardiovascular disease. Current interest has focused on n-3 polyunsaturated fatty acids (PUFA) and vitamin D. Clinical trial results on this subject are contradictory in many aspects. Several studies indicate that n-3 PUFA consumption improves vascular and cardiac hemodynamics, triglycerides, and possibly endothelial function, autonomic control, inflammation, thrombosis, and arrhythmia. Experimental studies show effects on membrane structure and associated functions, ion channel properties, genetic regulation, and production of anti-inflammatory mediators. Clinical trials evaluating a possible reduction in cardiovascular disease by n-3 PUFA have shown different results. Supplementation of vitamin D is common regarding prevention and treatment of osteoporosis. But vitamin D also seems to have several effects on the cardiovascular system. Vitamin D deficiency appears to be related to an increase in parathyroid hormone levels and can predispose to essential hypertension and left ventricular hypertrophy, increased insulin resistance, and eventually to atherosclerosis and adverse cardiovascular events. Randomized prospective clinical trials are needed to determine whether vitamin D and omega-3 FA supplementation therapy should be recommended as a routine therapy for primary or secondary prevention of cardiovascular disease.

## 1. Introduction

There are different ways of preventing and treating cardiovascular disease. Besides drug therapy and lifestyle changing dietary modification and supplementation play an increasingly important role in the conservative treatment of cardiovascular disease. Current interest has focused on n-3 polyunsaturated fatty acids (PUFA) and vitamin D [1]. Their potential cardiovascular risk reduction has been subject of many studies. n-3 PUFA seems to play a role in the treatment of coronary artery disease (CAD), cardiac arrhythmias, and heart failure. There are indications that they can also be used as an addition to the standard therapy of hypertriglyceridemia and diabetes. The results of some clinical studies are promising concerning cardiovascular outcomes. The GISSI-P study, for example, has shown that in addition to medical therapy daily supplementation with omega-3 fatty acids (FA) can reduce cardiac and all-cause mortality in patients after myocardial infarction [2].

The vitamin D receptor (VDR) is expressed in most tissues. Bioactive vitamin D belongs to a group of secosteroid molecules which are traditionally associated with bone and calcium metabolism [3]. The human body can synthesize vitamin D under influence of sunlight exposure out of 7-dehydrocholesterol, which is the major source (80% to 90%) of this substance in humans under natural conditions [4]. Vitamin D may potentially affect the treatment and prevention of hypertensive vascular disease, coronary artery disease, cardiac arrhythmias, peripheral vascular disease, lipid metabolism, and diabetes mellitus. Accumulating epidemiologic evidence suggests that hypovitaminosis D may be associated with an increased risk of cardiovascular events [5, 6], and experimental data generally support the hypothesis that vitamin D has a protective role in cardiovascular health [7, 8].

This paper will examine the relevance of omega-3 FA and vitamin D in cardiology and will provide an update of clinical trial results. 

## 2. Dietary Sources of n-3 PUFA

Fish is the major food source of long-chain n-3 PUFA, including eicosapentaenoic acid (EPA), docosahexaenoic acid (DHA), and, in smaller amounts, docosapentaenoic acid (DPA), a long-chain n-3 PUFA metabolite of EPA [9]. The fact that the correlation between DPA levels and fish consumption is low suggests that DPA levels in humans are predominantly determined by endogenous metabolism rather than diet. Alpha-linolenic acid is a plant-derived n-3 FA, which cannot be synthesized in humans and so is an essential dietary fatty acid. ALA is found in some sorts of seeds, nuts, and their oils. Some reports suggest that ALA might have cardiovascular benefits as well as EPA and DHA, but further studies of ALA's effects are urgently needed. Biochemical pathways to convert ALA to EPA and EPA to DHA are limited in humans, so that EPA and DHA levels are primarily determined by direct dietary consumption.

There has been a discussion if fish consumption or fish oil supplementation should be preferred. In addition to long-chain n-3 PUFA, fish contains specific proteins, vitamin D, selenium, and other minerals and elements. Most studies of death caused by coronary heart disease in generally healthy populations evaluated fish consumption, not fish oil supplementation. Because of the other mentioned ingredients of fish besides n-3 PUFA, this policy is reasonable, and the consumption of fish should be preferred. For individuals who cannot consume sufficient amounts of fish, fish oil supplementation is an alternative.

## 3. Effects of n-3 PUFA on the Cardiovascular System

**Figure 1:**
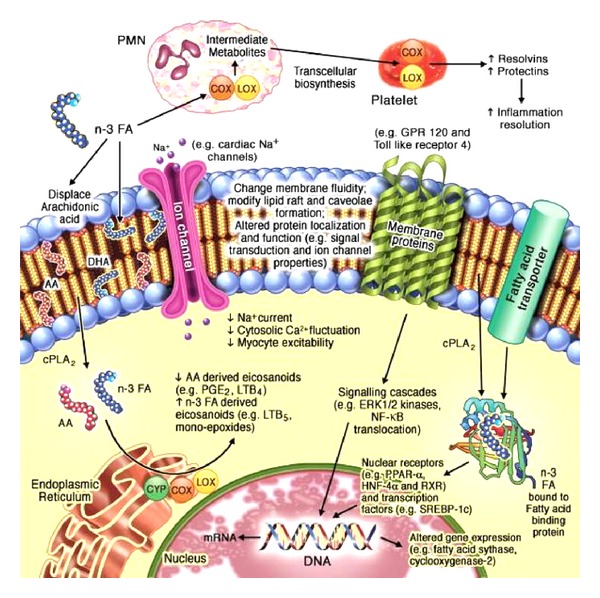
Molecular pathways affected by n-3 PUFA [9]. AA: arachidonic acid; COX: cyclooxogenase; cPLA_2_: cytosolic phospholipase A_2_; CYP450: cytochrome P450; DHA: docosahexaenoic acid; DNA: deoxyribonucleic acid; ERK: extracellular signal-regulated kinase; GPR 120: G-protein-coupled receptor 120; HNF: hepatic nuclear factor; LOX: lipoxygenase; mRNA: messenger ribonucleic acid; n-3 FA: n-3 fatty acids; NF-*κ*B: nuclear factor-kappaB; PGE2: prostaglandin E2; PMN: polymorphonuclear leukocyte; PPAR: peroxisome proliferator-activated receptor; RXR: retinoid X receptor; SREBP-1c: sterol regulatory element binding protein-1c.

The observed effects on cardiovascular risk factors are caused by the influence of n-3 PUFA on multiple relevant molecular pathways (Figure 1). Cellular and organelle functions are strongly influenced by membrane lipid environments. Lipid microdomains contribute to the modulation of numerous cellular functions like signal transduction, protein and membrane trafficking, and ion channel kinetics. The incorporation of n-3 PUFA in cell and organelle membrane structures alters their physicochemical properties and influences the localization, function, and signaling of membrane-associated proteins [9]. This might contribute to potential anti-inflammatory and antiarrhythmic effects.

In animal-experimental and *in vitro* studies, n-3 PUFA directly affected myocyte electrophysiology altering the function of membrane sodium channel, L-type calcium channel, and sodium-calcium exchanger. This might contribute to reduced myocyte excitability and cytosolic calcium fluctuations especially in ischemic or damaged cells. However, specific effects in experimental studies have not always been consistent.

Also some clinical trials for arrhythmia and sudden death after ischemia suggest that omega-3 FA has a direct electrophysiological effect on the myocardium. It is hypothesized that during ischemia, a reduction in the sodium ion current protects hyperexcitable tissue, and a reduction in the calcium ion current reduces arrhythmogenic depolarizing currents [10]. Whereas accumulating evidence suggests that incorporation of n-3 PUFA into and resultant changes in lipid membranes contributes to effects on ion channels, and that n-3 PUFA might also directly interact with membrane channels and proteins, the potential relevance of these experimentally observed influences on ion channels to health effects on humans is not fully established.

n-3 PUFAs are natural ligands of several nuclear receptors and transcription factors regulating gene expression in multiple tissues [11, 12]. These receptors regulate cellular functions related to cardiovascular disease, including lipid metabolism, glucose-insulin homeostasis, and inflammation. n-3 PUFA can also affect activation of transcription factors.

Another anti-inflammatory effect may be contributed to the lowering of arachidonic acid-(AA-) derived eicosanoids caused by n-3 PUFA consumption [13, 14].

Some n-3 PUFA-derived eicosanoids, in contrary, have an anti-inflammatory effect and seem to play a role in cardiovascular protection [15].

## 4. Benefits of n-3 PUFA Consumption for the Cardiovascular System

**Figure 2:**
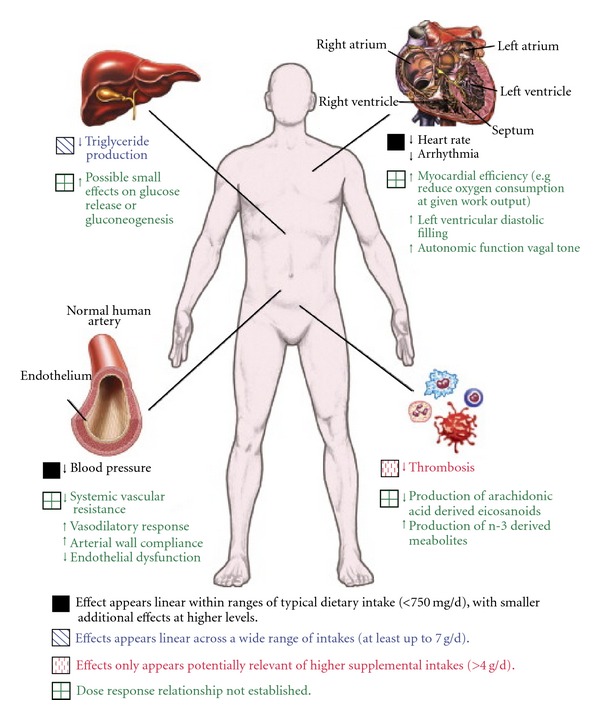
Physiological effects of n-3 PUFA that might influence cardiovascular disease (CVD) risk [9].

Physiological effects of n-3 PUFA that might influence the risk of cardiovascular disease (CVD) are depicted in Figure 2. Randomized trials assessed the efficacy of fish oil supplementation in a population after myocardial infarction (MI) with an ejection fraction of 40%. The GISSI-Prevention (GISSI-P) study randomized 11,324 patients to n-3 PUFA within three months after MI. Results demonstrated relative risk reductions in overall mortality (20%), cardiac mortality (30%), and sudden cardiac death (SCD, 45%) with 1 g/day of highly purified omega-3 acid ethyl esters (Omacor) over a 3.5-year period [2]. Significant benefits emerged within three to four months especially in those with extensive left ventricular dysfunction. Some studies suggest that n-3 PUFA improves heart rate variability as a marker of autonomic function [16, 17]. Holter monitor recordings showed an increase in heart rate variability (HRV) in the fish oil group [18]. Observational studies have linked omega-3 FA with the prevention of sudden cardiac death (SCD). Heart rate variability has been shown to be one of the best predictors of the risk of SCD. The data showed clearly that n-3 fatty acids increase HRV. This supports the hypothesis that an increased intake of n-3 fatty acids may reduce the risk of SCD [19]. However, in a larger cohort assessed in the Japan EPA lipid intervention study (JELIS) there was lack of an effect of fish oil on the incidence of sudden death and heart rate variability [20]. 

Atrial fibrillation (AF) is the most common sustained arrhythmia and is associated with considerable morbidity and mortality [21]. The role of omega-3 FA and its potential antiarrhythmic effect in the prevention of AF have been postulated. AF is a common complication after coronary artery bypass grafting operation (CABG).A prospective randomized study showed the efficacy of fish oil on AF after CABG. Perioperative intravenous infusion of polyunsaturated fatty acids (PUFA) reduced the incidence of AF after CABG leading to a shorter stay in the intensive care unit (ICU) and in hospital [22]. However, outcome of clinical research has been contradictory, and a definite role of omega-3 FA in the setting of AF has not been demonstrated [23]. The results of the OMEGA, a randomized, placebo-controlled trial to test the effect of highly purified omega-3 FA on top of modern guideline-adjusted therapy after myocardial infarction, showed a low rate of SCD, total mortality, and major adverse cerebrovascular and cardiovascular events within 1 year of followup after guideline- adjusted treatment and secondary prevention of acute myocardial infarction [24]. The results of this study should be interpreted carefully, because it was substantially underpowered. Alpha omega (study of omega-3 fatty acids and coronary mortality) studied n-3 PUFA supplemented margarine in post-MI patients [25]. The study outcome was neutral, the study was underpowered as well.

GISSI-heart failure (GISSI-HF) was a large adequately powered trial evaluating n-3 PUFA in heart failure [26]. Results demonstrated a significant and clinically important reduction of all-cause mortality with 1 g Omacor in a heart failure population. n-3 PUFA supplementation showed an absolute risk reduction of 1.8% in all-cause mortality seen over two years of followup. There was little benefit in atherothrombotic events, such as MI or stroke. 

Another effect of n-3 PUFA is lowering of plasma triglycerides [27]. This effect is caused by several mechanisms like reduced hepatic very low-density lipoprotein synthesis, increased fatty acid beta-oxidation, reduced delivery of nonesterified fatty acids to the liver, reduced hepatic enzyme activity for triglyceride synthesis, and increased hepatic synthesis of phospholipids rather than triglycerides.

n-3 PUFA consumption reduces heart rate and systolic and diastolic blood pressure [28, 29]. The decrease of heart rate is probably caused by direct effects on electrophysiologic pathways [10] and by indirect effects like the improving of left ventricular diastolic filling or an augmented vagal tone [16]. According to some trials the reduction of blood pressure is a consequence of increased nitric oxide production, a mitigated vasoconstrictive response to norepinephrine and angiotensin II, enhanced vasodilatory responses, and improved arterial compliance [30, 31].

The suspected antithrombotic effects of n-3 PUFA seen in some studies could not be confirmed in some human trials, where n-3 PUFA had no relevant effect on platelet aggregation and coagulation factors [32, 33].

Several trials found an improvement of endothelial function in some studies decreased markers of endothelial dysfunction were measured after n-3 PUFA consumption [34]. The normalization could partly mediate a protective effect against CVD.

In some studies n-3 PUFA consumption improved cardiac filling and myocardial efficiency measured by a reduced workload-specific oxygen demand [35]. In two trials n-3 PUFA even improved left ventricular function in patients with heart failure [36, 37].

The trial results concerning the influence of n-3 PUFA on insulin resistance and diabetes are still contradictory. While some trials found a modestly higher incidence of type 2 diabetes caused by n-3 PUFA consumption, most of the trials did not see any consistent effects on fasting glucose or hemoglobin A1c in patients with non-insulin-dependent diabetes [38].

Because of the above-mentioned suspected anti-inflammatory properties of n-3 PUFA, fish oil is already used as an adjunctive therapy for several inflammatory diseases like rheumatoid arthritis [39]. Nevertheless, the clinical impact of such anti-inflammatory effects, especially at usual dietary doses, is still unclear and will have to be subject of further studies. Possible effects of n-3 PUFA in the cardiovascular system are listed in Table 1.

According to numerous meta-analyses, all these possible benefits of n-3 PUFA seem to lead to a significant reduction of mortality by chronic heart disease (CHD) [40]. 

## 5. Potential Adverse Effects of n-3 PUFA Treatment

Adverse effects of omega-3 FA were also documented. The food and drug administration (FDA) product label on Lovaza (omega-3-acid ethyl esters) warns against potential bleeding complications when administered in combination with anticoagulants [41]. This warning is based on observational studies that suggested a prolonged bleeding time in populations ingesting high levels of fish oil [42]. On the other hand, in randomized clinical trials of patients undergoing coronary artery bypass graft surgery, percutaneous transluminal coronary angioplasty, and endarterectomy and diagnostic angiography, no adverse bleeding-related events have been demonstrated [43]. However, no serious adverse events in patients receiving antiplatelet and anticoagulant therapy in addition to fish oil supplementation have been reported. 

Besides, there have been concerns about possible contaminants present in some fish species, like methylmercury, dioxins, and polychlorinated biphenyls (PCBs). The concentration of these contaminants in some fish species has been under examination in the USA [9]. In most fish species, mercury levels are quite low, only in selected few species they were moderate or even near the US. Food and Drug Administration action level of 1 *μ*g/g (e.g., tilefish, swordfish, shark, and Mexico King mackerel). Mercury exposure levels common in the USA have no relation to higher CVD risks [9]. PCB and dioxin levels are usually low in commercially sold fish and according to the results of one US analysis contribute approximately 9% to total dietary exposure. So for general adult population the health benefits of modest fish consumption significantly overweigh the potential risks. There are special recommendations for sensitive subpopulations like young children and women of childbearing age.

## 6. Dietary Guidelines

There are several dietary guidelines for FA [44]. In these guidelines there is convergence recommending at least 250 mg/day EPA + DHA or at least 2 servings/week of fish, preferably oily fish. For pregnant women, nursing mothers, and young children, these recommendations are modified. The American Heart Association 2020 Strategic Impact Goals defined consumption of at least 2 3.5-oz servings/week of fish, preferably oily fish, as one of five primary dietary metrics that characterized ideal cardiovascular health [45]. The 2010 US. Dietary Guidelines for Americans recommended for individuals with higher and average CVD risk 2 4-oz seafood servings/week, which should provide an average of at least 250 mg/day EPA + DHA (1,750 mg/week) [46]. At present in most countries EPA + DHA intakes are much lower than recommended.

## 7. Sources of Vitamin D and Risk Factors for Vitamin D Deficiency

There are two forms of vitamin D: Vitamin D_2_ (ergocalciferol) is an ultraviolet B (UVB) radiation product of ergosterol. It can be consumed as supplement or in fortified foods. Vitamin D_3_ (cholecalciferol) is a product of UVB radiation of 7-dehydrocholesterol, is synthesized in the human epidermis, and can be consumed in oily fish, fortified foods, or a supplement. In the liver vitamin D is converted to 25(OH)D, which is the major circulating metabolite and which should be measured to clinically assess vitamin D status. In the kidney 25(OH)D is converted to its active form, 1,25(OH)_2_D, which can be regarded as a hormone, because it is produced primarily in one organ, the kidney, and then circulated throughout the body, where it exerts several important effects. It crosses the cell membrane and cytoplasm and reaches the nucleus, where it binds to the vitamin D receptor (VDR), which is present in most tissues including endothelium, vascular smooth muscle, and myocardium. Directly or indirectly, 1,25(OH)_2_D regulates more than 200 genes, including those involved in renin production in the kidney, insulin production in the pancreas, release of cytokines from lymphocytes, and growth and proliferation vascular smooth muscle cells as well as cardiomyocytes [6, 47].

The synthesis of vitamin D_3_ in response to UVB radiation in sunlight depends on many factors, including latitude, altitude, time of year and day, weather, age, skin pigmentation type, clothing, activity, and other aspects of the environment like air pollution. Modern human cultures produce less vitamin D cutaneously, in part because of increasingly indoor lifestyles and the avoidance of sun exposure by using sunscreen and other strategies. Sunscreen with a sun protection factor of 15 blocks approximately 99% of cutaneous vitamin D production. Other factors associated with vitamin D deficiency are poor nutrition, chronic illness, and renal failure.

Risk factors for vitamin D deficiency are pointed out in Table 2.

## 8. Effects of Vitamin D and Vitamin D Deficiency on the Cardiovascular System

The ubiquity of the VDR and tissue 25(OH)D-1*α*-hydroxylase provides insight into several pathobiologic pathways through which hypovitaminosis D may mediate vascular health. The VDR knockout mouse, an animal model emulating vitamin D deficiency, displays increased blood pressure, serum angiotensin, and tissue renin [48]. In *in vitro* studies vitamin D analogs directly suppress renin gene expression via a vitamin D response element that is present in the renin gene [48]. Vitamin D analogs have been shown to inhibit the production of several proinflammatory Th-1 cytokines, such as IL-2 and IFN-*γ*, while stimulating the effects of Th-2 lymphocytes, leading to a reduction in matrix metalloproteinase and reducing plaque production and instability. Furthermore, it has been shown to have immunosuppressive effects reducing the proliferation of lymphocytes and the production of cytokines, which have been identified as playing an important role in atherogenesis. VDR agonists have also been shown to downregulate plasminogen activator inhibitor 1 in human aortic smooth muscle cells but not endothelial cells. Besides, vitamin D seems to have a dose-dependent role in the inhibition of vascular calcification [5].

Chronic kidney disease (CKD) has been recognized as a powerful cardiovascular risk factor. Agarwal et al. found that the administration of an activated vitamin D analog (paricalcitol) reduced proteinuria in 51% of the 57 patients with CKD in comparison with only 25% of the 61 study participants who received placebo (*P* = 0.004), suggesting a direct vascular effect of vitamin D [49]. Some observational studies have reported an increased risk for death in patients with CKD and low serum 25(OH)D levels. Other studies have shown an association between treatment with activated vitamin D and lower all-cause and cardiovascular mortality in patients with CKD.

## 9. Benefits of Vitamin D for the Cardiovascular System

A wide range of cardiovascular diseases has been associated with vitamin D deficiency involving multiple potential mechanisms. Essential hypertension is a major risk factor for cardiovascular disease [3]. Vitamin D appears to be related to blood pressure control via multiple pathways and is inversely related to serum renin activity [48]. The results of clinical studies have shown that vitamin D levels were inversely associated with systolic and diastolic blood pressure, and vascular resistance [50, 51]. Conversely, in another study there was no association between vitamin D levels in newly diagnosed patients with hypertension and their matched controls [52]. The NHANES III study also observed the correlation between vitamin D levels and peripheral vascular disease. Low vitamin D levels were associated with a higher prevalence of peripheral arterial disease [53]. Vitamin D deficiency is also strongly associated with increased thickness of the intima-media in carotid arteries among type 2 diabetic patients [54]. 

Many coronary risk factors, including hypertension, diabetes mellitus, and lipid levels are affected by vitamin D: Multiple studies evaluated the relation of vitamin D prospectively with long-term cardiovascular outcomes in subjects without history of cardiovascular disease [3]. In healthy males between the ages of 40 and 75 years without history of coronary artery disease, vitamin D deficiency was associated with an increased rate of myocardial infarction over a 10-year period [55]. Similarly, in the Framingham Offspring study subjects without history of cardiovascular disease and vitamin D deficiency had increased risk for developing a first-cardiovascular event after 5 years of followup compared with subjects with optimal vitamin D levels (>15 ng/mL) [56]. Vitamin D can also indirectly affect cardiac function by altering parathyroid hormone and serum calcium levels [3]. It has been noted that osteoporosis, osteopenia, and low vitamin D levels are common in patients with congestive heart failure [57]. Observational studies have shown that there is ethnic variation in the incidence of heart failure and serum vitamin D levels. For example, vitamin D deficiency and hyperparathyroidism are more common in African American patients, and heart failure in this population is characterized by greater incidence, disease severity, and overall mortality compared to other populations [58–61]. 

**Figure 3:**
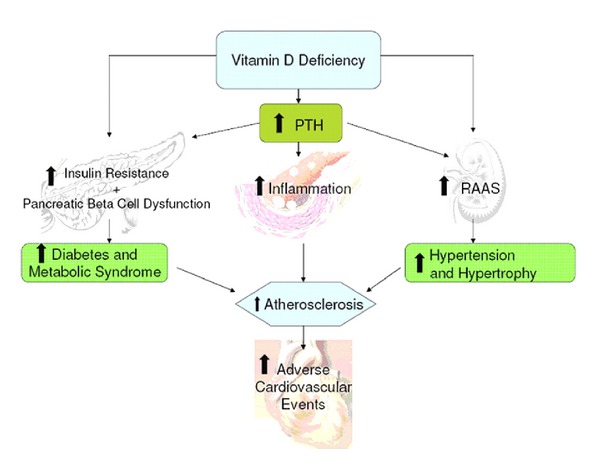
Possible mechanism of increased cardiovascular (CV) risk from vitamin D deficiency. PTH: parathyroid hormone; RAAS: renin-angiotensin-aldosterone system.

Possible mechanisms of increased cardiovascular (CV) risk from vitamin D deficiency are shown in Figure 3. Vitamin D deficiency activates the renin-angiotensin-aldosterone system, can predispose to hypertension and left ventricular hypertrophy. It causes an increase in parathyroid hormone, which increases insulin resistance and is associated with diabetes, hypertension, inflammation, and increased cardiovascular risk [6] (Table 3). 

Multiple, recent studies have shown an association between vitamin D deficiency and a variety of adverse cardiovascular outcome parameters [62–64].

A meta-analysis of 18 randomized controlled trials comprising 57,000 individuals showed that a vitamin D intake >500 IU/day improved all-cause mortality, in part by decreasing cardiovascular (CV) deaths [65]. Data regarding both efficacy and CV safety for vitamin D appear to be superior to that for calcium supplements.

Currently, most experts define vitamin D deficiency as a 25(OH)D level of <20 ng/mL (50 mmol/L) and vitamin D insufficiency as 21 to 29 ng/mL. The optimal concentration of 25(OH)D is at least 30 ng/mL [66]. 

## 10. Potential Adverse Effects of Vitamin D Treatment

Excessive use of vitamin D supplements can cause progressive accumulation and toxic effects, presenting as hypercalcemia and renal damage. Clinical symptoms include anorexia, nausea, and vomiting, followed by polyuria, polydipsia, pruritus, and eventually even renal failure [3]. As to the present knowledge such toxic effects occur only with prolonged (at least several months) daily intake of more than 1,000 *μ*g (40,000 IU). Excessive sunlight exposure cannot cause vitamin D toxicity because UVB converts excess vitamin D_3_ to biologically inert isomers.

## 11. Dietary Guidelines

Up to 95% of the body's vitamin D requirement comes from the synthesis in the epidermis in response to sun exposure. The remaining 5% are ingested from dietary sources. Studies indicate that the average U. S. adult consumes approximately 230 IU vitamin D per day, whereas an estimated daily intake of 1,000 to 2,000 IU would be necessary to satisfy the body's needs for most people.

The recommended dosage of supplemental vitamin D has increased within the last years. Currently US Government recommends 600 IU daily for children and adults up to the age of 70 years and 800 IU for seniors above 70 years [67]. But according to some experts this dosage is still too low and should be increased up to 5,000 IU for healthy adults and 6,000 IU for pregnant women and lactating mothers. The tolerable upper intake level (UL) is set at 4,000 IU per day by the US Government, but according to many experts should be raised to 10,000 IU per day.

## 12. Future Prospects

There are several trials underway to fill some gaps in the understanding of intervention with n-3 PUFA and vitamin D in cardiovascular disease.

The ongoing vitamin D and omega-3 trial (VITAL) is a large randomized, double-blind, placebo-controlled, 2 × 2 factorial trial of vitamin D and marine omega-3 FA supplements in the primary prevention of cancer and cardiovascular diseases (CVD) among a multiethnic population of 20,000 US men aged ≥50 and women aged ≥55. Vitamin D is used in the form of vitamin D3 (cholecalciferol) and 2,000 IU/day and omega-3 FA is used as Omacor fish oil, eicosapentaenoic acid (EPA) + docosahexaenoic acid (DHA), 1 g/day. The mean treatment period will be five years the study is expected to be completed in 2016. Baseline blood samples are collected in at least 16,000 participants, with followup blood collection in about 6,000 participants. Yearly followup questionnaires will assess treatment compliance, use of nonstudy drugs or supplements, occurrence of endpoints, and cancer and vascular risk factors. Self-reported endpoints will be confirmed by medical record review by physicians blinded to treatment assignment, and deaths will be ascertained through national registries and other sources. Ancillary studies are planned to investigate the effect of vitamin D and omega-3 FA on other multiple diseases like for example diabetes, glucose intolerance, and hypertension. The results of VITAL are expected to influence individual decisions, clinical recommendations, and public health guidelines regarding the use of vitamin D and marine omega-3 FA supplements for the primary prevention of cancer and CVD [68]. 

GISSI-R&P was launched in 2004 and is an ongoing large-scale, randomized-controlled trial conducted in Italian general practice to assess the efficacy and safety of omega-3 PUFAs in reducing cardiovascular mortality (including sudden death) and hospitalization for cardiovascular reasons in patients at high CVD risk but without history of myocardial infarction. The secondary epidemiological aim is to assess the feasibility of adopting current guidelines in everyday practice in order to optimize all available preventive strategies in people at high cardiovascular risk.

A study of cardiovascular events in diabetes (ASCEND) is a randomized study, which should provide reliable evidence about the effects of aspirin and of omega-3 FA on types 1 and 2 diabetes. It is scheduled to continue until 2017.

 The omega-3 fatty acids for the prevention of post-operative atrial fibrillation (OPERA) is a large, randomized-controlled trial, which is expected to complete in late 2012. It investigates the effects of omega-3 PUFAs on the major public health challenge of atrial fibrillation.

## 13. Conclusion

The systematic analysis of the currently available literature in this paper could not prove a significant benefit of a general vitamin D or omega-3 FA supplementation for the treatment and prevention of CVD, as the trial results are still contradictory in many aspects. In addition, the adequate dosage of these substances remains unclear. 

Several studies indicate that n-3 PUFA consumption improves vascular and cardiac hemodynamics, triglycerides, and possibly endothelial function, autonomic control, inflammation, thrombosis, and arrhythmia. Experimental studies show relevant effects on membrane structure and associated functions, ion channel properties, genetic regulation, and production of anti-inflammatory mediators. Clinical trials evaluating a possible reduction in CVD by n-3 PUFA have shown different results. Nevertheless, as part of a healthier dietary pattern including fruits, vegetables, whole grains, nuts, vegetable oils, and dairy the consumption of fish would be another reasonable contribution and should be recommended.

Supplementation of vitamin D is common, regarding the “traditional” roles of vitamin D with its positive effects on bone mineral density, for the prevention and treatment of osteoporosis. Furthermore, the individual risk of vitamin D deficiency has to be taken into account. “Nontraditional” roles of vitamin D seem to be malignancies like colorectal cancer, diabetes mellitus, multiple sclerosis, impaired immune response, and several effects on the cardiovascular system. In which individuals a supplementation of vitamin D is reasonable, and in which cases serum hydroxyvitamin D level should be measured before is still unclear.

Randomized prospective clinical trials are needed to determine whether vitamin D and omega-3 FA supplementation therapy could have any potential benefit in reducing future CVD events and mortality risk, and if it should be recommended as a routine therapy for primary or secondary prevention of cardiovascular disease. 

## Figures and Tables

**Table 1:** 

Potential effects of n-3 PUFA	
Antiarrhythmic effects	
Improvement of autonomic function	
Decreased platelet aggregation	
Vasodilation	
Decreased blood pressure and heart rate	
Anti-inflammatory effects	
Improvements in endothelial function	
Reduced triglycerides	
Improvement of vascular and cardiac hemodynamics	

**Table 2:** 

Risk factors for vitamin D deficiency	
Elderly	
Darkly pigmented skin	
Institutionalized of homebound	
Increased distance from equator	
Winter season	
Cover-up clothing and/or sunscreen	
Air pollution	
Smoking	
Obesity	
Malabsorption	
Renal disease	
Liver disease	
Medications: anticonvulsants, glucocorticoids, antirejection, and immunodeficiency virus medications	

**Table 3:** 

Potential effects of vitamin D deficiency on the cardiovascular system	
Activation of renin-angiotensin-aldosterone system	
Predisposition to hypertension and left ventricular hypertrophy	
Increase in parathyroid hormone	
Increase in insulin resistance	
Association with diabetes, hypertension, and inflammation	
Increase in lipid levels	
Increased thickness of the intima-media in carotid arteries	
Increased rate of myocardial infarction	
Decrease of cardiac function	
